# Psychosocial factors associated with alcohol use in lower socioeconomic position populations: a scoping review

**DOI:** 10.1186/s12889-025-24508-z

**Published:** 2025-10-31

**Authors:** Sarah Dance, Sally Adams, Andrew Weyman, Amy Herbert, Chloe Burke, Netanya Cassidy, Nina Higson-Sweeney, Charlotte Dack

**Affiliations:** 1 Department of Psychology, University of Bath, Bath, BA2 7AY UK; 2 School of Psychology, University of Birmingham, Birmingham, B15 2TT UK; 3 Department of Experimental Psychology, University of Oxford, Oxford, OX2 6GG UK

**Keywords:** Alcohol, Inequality, Intervention, Socioeconomic position, Mental health, Stress, Availability

## Abstract

**Background:**

Improved understanding of psychosocial factors associated with alcohol use in lower socioeconomic position (SEP) populations could inform theory and practice in the development of interventions aimed at reducing alcohol-related harm in this population. This review aimed to review and synthesise the literature on these associations for lower SEP populations.

**Methods:**

We conducted a scoping review of studies examining associations between psychosocial factors and alcohol use in lower SEP populations. Web of Science (Core Collection), Scopus, Embase (Embase and Medline), PubMed, and APAPsycNet (PsycInfo) were searched. Out of 6597 identified articles, 26 articles were included from the databases. Hand searching of references of these studies identified four additional eligible studies. Narrative synthesis was used to synthesise identified factors.

**Results:**

30 studies in total (21 quantitative, nine qualitative) were included. Identified psychosocial factors related to mental health, stress, drinking motives, alcohol availability, adolescence, cognitive factors, and other psychosocial factors.

**Conclusions:**

The array of identified psychosocial factors can inform future directions for tailored alcohol interventions for lower SEP populations. The evidence base is predominantly comprised of quantitative studies investigating factors such as mental health and stress. Future research in the area would benefit from greater use of qualitative studies to complement these insights and generate improved understanding of experiences of alcohol use among lower SEP populations. Individual-level drinking motivations (e.g., coping with stress and mental health) may be amplified by the environmental context of disadvantaged neighbourhoods, which may play a role in disposition to drinking as a coping mechanism. Yet, these communities may have reduced access to alternative coping resources. Policymakers implementing population-level interventions to reduce alcohol availability may also need to consider what and how alternative coping resources and strategies for stress and mental health can be implemented in underserved communities. Targeting specific drinking reasons may increase intervention acceptability among lower SEP populations.

**Supplementary Information:**

The online version contains supplementary material available at 10.1186/s12889-025-24508-z.

## Background

Alcohol use (AU) is linked to three million deaths and more than 200 health conditions each year worldwide [1]. However, alcohol harms are not equally distributed. The alcohol harm paradox is where lower socioeconomic position (SEP) populations exhibit higher rates of alcohol-related ill health and mortality, despite evidence of lower consumption rates than higher SEP populations [2] (predominantly based on evidence from high-income countries [3, 4]). This suggests that socioeconomic differences in health impacts cannot be explained by consumption rates alone [3]. Therefore, tailored interventions for lower SEP populations may need to account for contextual variables.

Most alcohol public health interventions focus on reduced AU in non-targeted approaches focused on the general population. However, reduced participation in public health campaigns (e.g., Dry January) among lower SEP populations [5] may suggest reduced impact in this group, whereas legislative action such as minimum unit pricing (MUP) demonstrates greater impact in reducing alcohol-related harms among lower SEP populations [6]. Enhanced understanding of the psychosocial context of AU in lower SEP populations may increase the impact of tailored public health interventions, in line with Medical Research Council guidance on intervention design [7] and as demonstrated by tailored public health interventions [8, 9]. However, only one intervention published in English, from the UK, aimed at reducing AU has been specifically developed for lower SEP populations. This text-messaging intervention did not significantly reduce binge drinking, but high levels of engagement were observed [10]. Therefore, there may be scope to investigate the potential for a tailored approach among this population.

Epidemiological [3, 11–13] and sociological [14–16] literature has focused on how SEP populations may differ in terms their AU, drinking patterns, and alcohol-related consequences. The literature on contextual psychosocial factors associated with AU tends to focus on associations within younger populations, such as adolescents [17, 18] and university students [19, 20]. For example, low self-esteem, stress, anxiety [20], and social norms [19] have been associated with greater disposition to drinking in student populations. It is unclear to what extent factors associated with drinking in these populations may apply to other groups. A systematic review of proposed hypothetical and empirical explanations to explain the alcohol harm paradox identified a range of variables, ranging from individual (e.g., biological impact of multiple health risks) to structural (e.g., corporate influence on policy decisions) explanations. Risk behaviours, such as drinking patterns, clustered health behaviours (e.g., a combination of tobacco and alcohol and other drug use, as well as low physical activity levels), type of alcoholic drink, and alcohol quantity consumption history, were the most common explanations for the paradox [4]. In support, a systematic review of socioeconomic differences in mortality suggests that heavy episodic drinking explains approximately 15–30% of socioeconomic inequalities in mortality [3]. Other proposed explanations included individual explanations (e.g., stereotypes may lead individuals to exhibit behaviours others assume of them), contextual explanations (e.g., reduced social support), social disadvantage explanations (e.g., reduced access to services), and structural explanations (e.g., occupational working conditions) [4]. However, a broader literature on psychosocial factors associated with AU in lower SEP populations (beyond proposed explanations of the paradox) has yet to be synthesised. In particular, it has not been synthesised from a health psychology perspective informed by a psychosocial approach [21], with a focus on identifying targets to inform interventions. Interventions accounting for the psychosocial context of a health behaviour tend to have increased effectiveness among target populations [7].

We conducted a scoping review to investigate psychosocial factors associated with AU in lower SEP populations. Scoping reviews are useful for outlining factors within a diverse research area [22].

## Methods

### Design

This scoping review mapped psychological and social factors associated with AU among lower SEP populations. A scoping review aligns with our aim to map concepts within a heterogeneous research area. It is also appropriate for examining a wider objective than a systematic review may do [22]. Our approach involved synthesising reported findings related to reported factors. This aligns with other scoping reviews which have mapped factors associated with a health-related concept and considered significance of reported findings [23–25]. Following relevant guidance [22], a protocol was pre-registered (https://osf.io/6574d) and the PRISMA Extension for Scoping Reviews (PRISMA-ScR) reporting guidelines [26] were followed (see Supplementary Materials).

### Inclusion criteria

Inclusion criteria aligned with the Population-Concept-Context (PCC) framework; a recommended framework for scoping reviews [22]. The population was lower SEP populations. We included both studies which focused on a lower SEP population, and studies which compared lower and higher SEP populations. Where a spectrum of SEP was reported, we used the lower range as lower SEP. In order to scope the existing literature, we considered lower SEP broadly and included studies with measurements such as income, education, occupation, and community disadvantage. This approach was informed by nationally used measurements of socioeconomic status (SES) and social class in the UK, including occupation [27] and social grade based on qualifications and occupation [28]. We were also informed by the index of multiple deprivation, which incorporates several measures (e.g., income, employment, education) and is a nationally used measurement in the UK to assess alcohol-related inequalities [29]. We excluded studies which focused on health inequalities in some populations, such as populations experiencing homelessness, refugee populations, immigrant populations, and indigenous populations. Whilst these populations may experience social disadvantage, we did not consider these populations as part of a lower SEP population specifically for the purposes of this review. We also excluded studies which used a lower- or middle-income country as their unit of analysis for SEP, although studies from a lower- or middle-income country which defined a lower SEP population according to the criteria above (e.g., based on measurements such as income, education, occupation, and community disadvantage) would have been eligible for inclusion. We included studies with adult populations aged 18 years old or above (or a mean average age of at least 18 years for studies with participants above and below 18 years). We included only adult populations because we anticipated that there may be distinct psychosocial factors associated with AU in adolescent populations and to inform interventions targeted towards AU in adulthood.

The concept was psychosocial factors associated with AU. We sought to identify psychological factors (e.g., mental health) or social factors (e.g., alcohol availability). Associated factors could be referred to in studies by terms such as an association (quantitative studies) or related concept (qualitative studies). In this review, we defined psychosocial factors according to the biopsychosocial model. This model considers health more broadly than the biomedical model of health and disease, and proposes that psychological (e.g., factors related to an individual) and social (e.g., factors related to their social and physical environment) factors also play a role in health experiences and diseases [21].

The context was any setting or location. We included quantitative and qualitative studies of any design in English.

### Search strategy

**Fig. 1:**
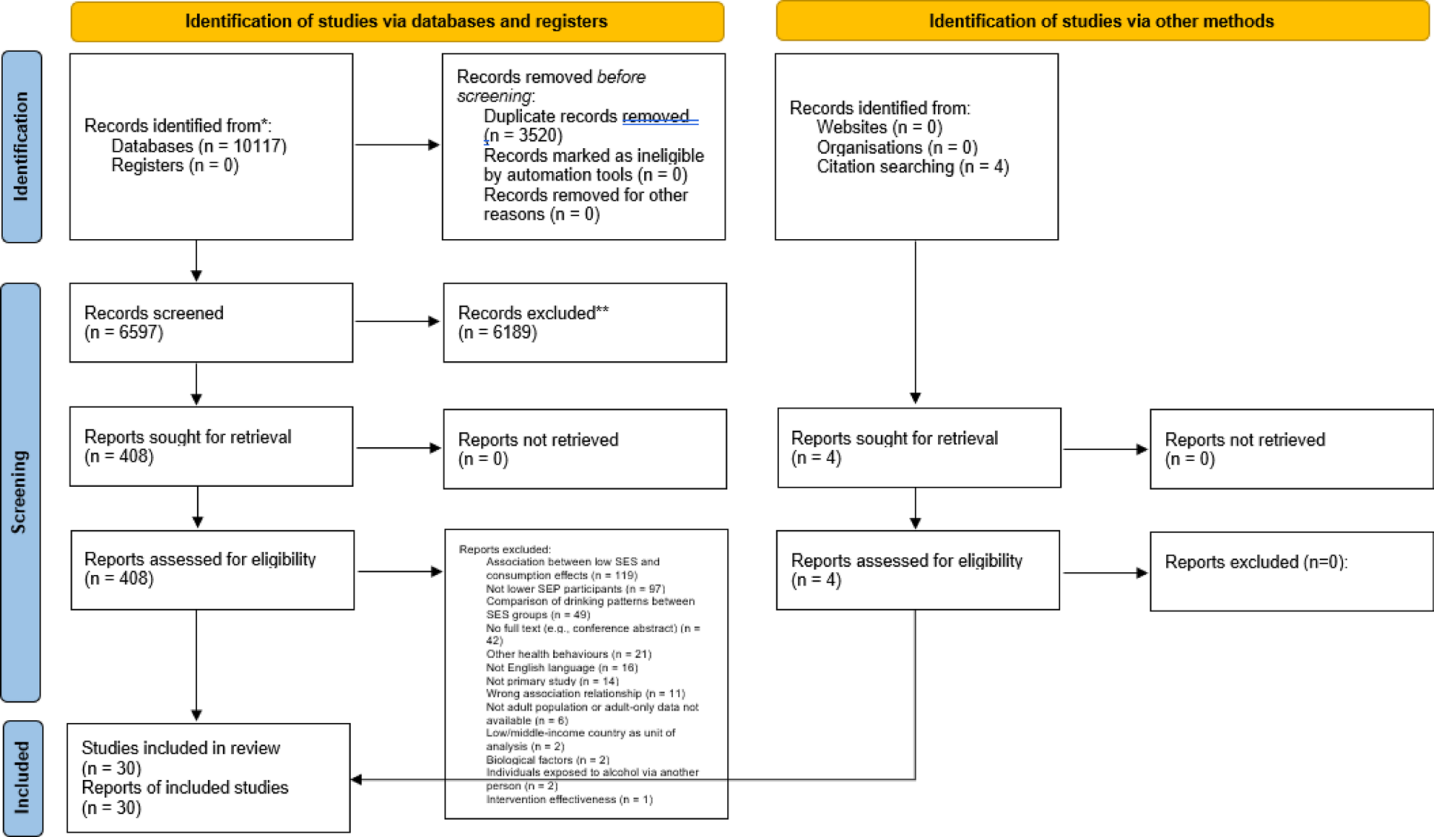
PRISMA flow chart

Search terms were developed to capture broad references to SEP and psychosocial factors (see Supplementary Materials for full search strategy). Five databases were searched: Web of Science (Core Collection), Scopus, Embase (Embase and Medline), PubMed, and APAPsycNet (PsycInfo). Databases were searched from January 2000 – December 2022, with a search update performed on 6th January 2025. Articles were downloaded into Endnote and imported into Covidence for de-duplication. All articles were screened by one author and independently screened by a second reviewer at title/abstract and full-text stage (K = 0.36, K = 0.30). Differences were resolved through reviewer discussion. To supplement the database searches, reference lists of included studies were hand-searched for relevant references (see Fig. 1).

### Data extraction and charting

Data were extracted using a Microsoft Excel document by one author, which was reviewed for accuracy by a second reviewer. Extracted data included: study characteristics, design, sample, lower SEP measure, AU measure, analysis method, number of participants, psychosocial factor measure (quantitative studies), and qualitative and quantitative findings.

Study characteristics are presented in Table 1 and findings are presented in Tables 2, 3, 4, 5 and 6. A narrative summary of study findings is provided in categories of psychosocial factors investigated and methodological designs. Supporting statistics are provided for identified associations among lower SEP populations. When populations are reported for each study, we used the term used by the primary study to describe SEP populations. In order to meet the aim of the review, we mainly focus on findings for lower SEP populations rather than extensively discuss higher SEP populations.

**Table 1:** Study characteristics

Study	Country	Design	Sample	Dataset	SEP measure	Number of participants	Age of sample	Psychosocial factor	Measure of alcohol use
Arora et al. [54]	India	Qualitative - Focus groups	Residents in two states in India	N/A	SES	171	18–58	Social acceptance	N/A
Assanangkornchai et al. [30]	Thailand	Quantitative - Secondary analysis of repeated cross-sectional survey	Adults in Thailand	2014 Thai National Health Examination Survey	Wealth index and highest education level	13, 177	20+ (46.7 mean average)	Major depressive episode	Questionnaire (AUDIT-10)
Boniface et al. [50]	UK	Quantitative - Secondary analysis of cross-sectional survey	Residents in two boroughs of London	South East London Community Health study	Income, occupation, housing status, and education	1, 052	16+	Common mental disorder	Questionnaire (AUDIT-10)
Cerda et al. [33]	USA	Quantitative - Secondary analysis of repeated cross-sectional survey	Residents in Louisiana, Mississippi, and Alabama, USA before Hurricanes Katrina/Rita	Panel Study of Income Dynamics	Income, education	439	18–85 (41.4 mean average)	Exposure to hurricane-related stressors	Questionnaire (other)
Elliot and Lowman [49]	USA	Quantitative - Secondary analysis of longitudinal study	Respondents who completed both waves of the National Co-Morbidity Survey	National Co-Morbidity Survey	Income, education	4, 979	15–59 (33.1 mean average, 10.7 SD)	Psychological resources (locus of control, religiosity)	Questionnaire (other)
Grzywacz & Almeida [34]	USA	Quantitative - Repeated telephone interviews	Adults who had previously participated in the National Survey of Midlife Development in the United States	National Study of Daily Experiences	Education	802	25–74 (44.6 mean average, 13.4 SD)	1. Daily stress and stress pile-up 2. Negative affect	Questionnaire (other)
Hart [58]	Australia	Qualitative - Interviews	Young adults living in or around a suburb in Melbourne, Australia	N/A	Residence in disadvantaged area	Not reported	18–24	1. Family influence. 2. Memories and emotions during drinking.	N/A
Heim et al. [40]	UK	Quantitative - Cross-sectional survey	UK adults	N/A	Social grade	1, 639	18–75 (47.7 mean average, 14.7 SD)	Coping drinking motive	Questionnaire (AUDIT-10)
Järvinen [57]	Denmark	Qualitative - Interviews	Employees in a Danish company	N/A	Social class	30	24–65 (43 average)	Risk conception	N/A
Lunnay et al. [51]	Australia	Qualitative - Interviews	Midlife Australian women	N/A	Economic capital, social capital, cultural capital	27	45–64	1. Enjoyment. 2. Coping drinking motive	N/A
Lunnay et al. [53]	Australia	Qualitative - Interviews	Midlife Australian women	N/A	Economic capital, social capital, cultural capital	50	45–64	1. Coping drinking motive 2. Stress 3. Mental health benefits 4. Loneliness	N/A
Lunnay et al. [16]	Australia	Qualitative - Interviews	Midlife Australian women	N/A	Economic capital, social capital, cultural capital	50	45–64	1. Stress 2. Coping drinking motive 3. Loneliness	N/A
Martinez et al. [32]	Norway	Quantitative - Secondary analysis of two cross-sectional studies	Adults in Norway	The Health Study in Oppland and Hedmark, and the Oslo Health Study	Education	10, 872	40–45	Depressive symptoms	Questionnaire (other)
McCarthy et al. [45]	USA	Quantitative - Secondary analysis of longitudinal study	Adults from a study on the long-term course of adolescent alcohol and drug abuse	A longitudinal study of the long-term course of adolescent alcohol and drug abuse	Education, occupation	172	22.12 average (1.6 SD)	Alcohol expectancies	Questionnaire (other)
Molina de la Fuente et al. [56]	Spain	Qualitative - Participatory action	Residents in two districts in Madrid, Spain	N/A	District SES	26	39–78	Socialising	N/A
Molina de la Fuente et al. [55]	Spain	Qualitative - Participatory action	Residents in two districts in Madrid, Spain	N/A	District SES	26	39–78 (57 average)	Gambling	N/A
Monk et al. [41]	UK	Quantitative – Secondary analyses of two cross-sectional surveys	Adults in the UK	N/A	Social grade based on occupation	11, 700	18–85	1. Mental health 2. Drinking motives	Questionnaire (AUDIT-C)
Mulia & Zemore [31]	USA	Quantitative - Secondary analysis of cross-sectional survey	Adults in the USA	2005 U.S. National Alcohol Survey	Poverty status based on income	4, 080	18+	Psychological stress (depressive symptoms)	Questionnaire (other)
Pollack et al. [46]	USA	Quantitative – Cross-sectional surveys combined with datasets	Adults interviewed during one of the cross-sectional surveys	1. Stanford Heart Disease Prevention Program 1979–1990 2. 1980 census data 3. Alcohol availability records from California Department of Alcoholic Beverage Control	Neighbourhood deprivation	8, 197	25–74	Alcohol availability	Questionnaire (other)
Puddephatt et al. [48]	UK	Quantitative – Secondary analysis of cross-sectional survey	Adults in the UK	2014 Adult Psychiatry Morbidity Survey	Occupation, education, and housing tenure	1436	16+	1. Social support 2. Neighbourhood environment	Questionnaire (AUDIT-10)
Rocheleau [35]	USA	Quantitative - Secondary analysis of longitudinal study	Respondents to the National Longitudinal Study of Adolescent to Adult Health	National Longitudinal Study of Adolescent to Adult Health	Social class based on parental education and employment	3, 749	22–31	Working long and intense hours during adolescence	Questionnaire (other)
Rodriguez-Stanley et al. [42]	USA	Quantitative – Cross-sectional survey	Adults in the US	N/A	1. Socioeconomic status based on education, income, employment. 2. Subjective Social Status	412	18–85	Psychological distress	Questionnaire (other)
Sabia et al. [38]	France	Quantitative - Prospective cohort study	Employees of a French company	GAZEL cohort	Education, occupational position	4, 073	55–65	Cognitive performance	Questionnaire (other)
Segrin et al. [44]	USA	Quantitative - Cross-sectional survey combined with dataset	US adults	2015 American Community Survey	Neighbourhood disadvantage	1050	21–70 (43.2 mean average, 13.7 SD)	1. Social Support 2. Negative emotionality	Questionnaire (other)
Segrin et al. [43]	USA	Quantitative – Repeated cross-sectional survey combined with dataset	US adults	2018 American Community Survey	Neighbourhood disadvantage	618	21–65 (30.80 mean average, 9.81 SD)	Psychological distress	Questionnaire (AUDIT – 7 items)
Shortt et al. [37]	UK	Quantitative – secondary analysis of repeated cross-sectional survey	Individuals living in private households in Scotland	Scottish Health Survey 2008–2011	Income	24, 557	16+	Alcohol outlet density	Questionnaire (other)
Shuai et al. [39]	UK	Quantitative - Cross-sectional survey	Undergraduate students who reported past year hazardous drinking	N/A	Subjective social status	219	18–25 (19.84 mean average, 1.32 SD)	1. Exposure to aversive experience 2. Internalizing symptoms 3. Drinking to cope	Questionnaire (AUDIT-10)
Ward et al. [52]	Australia	Qualitative - Interviews	Midlife Australian women	N/A	Economic capital, social capital, cultural capital	50	45–64	1. Personal and family stressors 2. Coping drinking motive	N/A
Yaogo et al. [36]	France	Quantitative - Prospective longitudinal cohort study	Individuals who participated in TEMPO study	TEMPO cohort	Childhood family income	674	22–35	Adolescent alcohol repeated intoxication	Questionnaire (AUDIT-10)
Zhu et al. [47]	China	Quantitative - Cross-sectional survey	University students	N/A	Family economic hardship	513	17–22 (19.29 mean average, 5.11 SD)	1. Perceived discrimination 2. Impulsivity	Questionnaire (other)

AUDIT = Alcohol use disorder identification test. N/A = Not applicable. Questionnaire (other): survey or question(s) other than AUDIT used

**Table 2:** Quantitative studies investigating associations between alcohol use and psychosocial factors within a lower SEP population

	Mental health	Stress	Adolescence	Availability	Cognitive
**Assanangkornchai et al.** [30] Major depressive disorder	---				
**Cerda et al. **[33] Exposure to hurricane-related stressors		---			
**Grzywacz & Almeida** [34] Daily stress and stress pile up		✓			
**Martinez et al. **[32] Depressive symptoms	---				
**Mulia & Zemore** [31] Psychological stress (depressive symptoms)	✓				
**Rocheleau **[35] Working intense hours in adolescence			X		
**Sabia et al.** [38] Cognitive performance					✓
**Shortt et al. **[37] Alcohol outlet density				---	
**Yaogo et al. **[36] Repeated alcohol intoxication in adolescence			✓		
**Total**	**3**	**2**	**2**	**1**	**1**

✓ = Significant association. --- = Mixed findings. X = Non-significant association

**Table 3:** Specificity of associations between alcohol use and psychosocial factors to a lower SEP population, compared to higher SEP populations

	Mental health	Stress	Adolescence	Availability	Cognitive
**Assanangkornchai et al.** [30] Major depressive disorder	X				
**Cerda et al.** [33] Exposure to hurricane-related stressors		✓			
**Grzywacz & Almeida** [34] Daily stress and stress pile up		✓			
**Martinez et al. **[32] Depressive symptoms	X				
**Mulia & Zemore** [31] Psychological stress (depressive symptoms)	X				
**Rocheleau** [35] Working intense hours in adolescence			X		
**Sabia et al.** [38] Cognitive performance					✓
**Shortt et al.** [37] Alcohol outlet density				✓	
**Yaogo et al.** [36] Repeated alcohol intoxication in adolescence			X		
**Total**	**3**	**2**	**2**	**1**	**1**

✓ = Significant association only found among lower SEP populations. X = Significant association found among higher SEP populations

**Table 4:** Quantitative studies of investigations of psychosocial factors pathways associated with alcohol consumption in lower SEP populations

	Mental health	Drinking motives	Stress	Cognitive	Availability	Social - other	Psychological - other
**Elliot and Lowman** [49] Psychological resources [locus of control, religiosity] (psychological - other)							✓✓
**Heim et al. **[40] Coping drinking motive		✓					
**McCarthy et al. **[45] Alcohol expectancies (cognitive)				✓			
**Monk et al. **[41] Mental health (mental health) Drinking motives (drinking motives)	---	---					
**Pollack et al.** [46] Alcohol availability					X		
**Puddephatt et al. **[48] Social support (social – other) Neighbourhood environment (social – other)						✓✓	
**Rodriguez-Stanley et al. **[42] Psychological distress (mental health)	X						
**Segrin et al.** [44] Social support (social – other) Negative emotionality (mental health)	✓					X	
**Segrin et al.** [43] Psychological distress (mental health)	✓						
**Shuai et al. **[39] Exposure to aversive experience (stress) Internalising symptoms (mental health) Drinking to cope (drinking motives)	✓	✓	✓				
**Zhu et al.** [47] Impulsivity (psychological – other)							X
**Total number of factors**	**5**	**3**	**1**	**1**	**1**	**4**	**3**

✓ = Significant association. X = Non-significant association. --- Mixed findings. Note: Pollack et al. (2005) states ‘data not shown’ for this finding

**Table 5:** Quantitative studies investigating associations between alcohol use and lower SEP tested with a psychosocial factor adjusted for as a confounder

	Mental health	Social - other
**Boniface et al.** [50] Common mental disorder	X	
**Grzywacz & Almeida** [34] Negative affect	✓	
**Zhu et al.** [47] Perceived discrimination (social – other)		X
**Total**	**2**	

✓ = Significant association remains when adjusted for psychosocial factor as confounder. This indicates that the psychosocial factor may not contribute to the relationship. X = Significant association no longer remains when adjusted for psychosocial factor as confounder. This indicates that the psychosocial factor may contribute to the relationship

**Table 6:** Qualitative studies investigating psychosocial factors associated with alcohol consumption in lower SEP populations

	Drinking motives	Stress	Mental health	Psychosocial - other
**Arora et al.** [54] Social acceptance				*
**Molina de la Fuente et al.** [56] Socialising				*
**Molina de la Fuente et al.** [55] Gambling				*
**Hart **[58] Family influence, memories and emotions during drinking				**
**Järvinen **[57] Risk conception				*
**Lunnay et al. **[51] Enjoyment (psychosocial – other) Coping drinking motive (drinking motives)	*			*
**Lunnay et al.** [53] Coping drinking motive (drinking motives) Stress Mental health benefits (mental health) Loneliness (psychosocial – other)	*	*	*	*
**Lunnay et al.** [16] Stress (stress) Coping drinking motive (drinking motives) Loneliness (psychosocial – other)	*	*		*
**Ward et al.** [52] Personal and family stressors (stress) Coping drinking motive (drinking motive)	*	*		
**Total**	**4**	**3**	**1**	**9**

* = Indicates factor identified in qualitative study

## Findings

### Search results and study characteristics

The searches identified 6597 articles after duplicate removal (5565 from the original search and 1032 from the updated search), of which 408 were screened at full text (see Fig. 1).

30 studies (21 quantitative, nine qualitative) were included (see Table 1). The studies originated from the following countries: USA (*n* = 10), UK (*n* = 6), Australia (*n* = 5), France (*n* = 2), Spain (*n* = 2), and one study each from China, Denmark, India, Norway and Thailand.

### Quantitative studies

#### Associations between alcohol use and psychosocial factors in lower SEP populations

Nine studies investigated associations between AU and psychosocial factors in lower SEP populations (see Tables 2 and 3).

##### Mental health

Three quantitative studies investigated associations between mental health and AU in lower SEP populations. One study investigated major depressive episode (MDE) [30] and two studies investigated depressive symptoms [31, 32]. Assanangkornchai et al. [30] found a significant association between MDE and harmful-dependent AU among the lowest (adjusted odds ratio (OR) = 7.14, 95% confidence interval (CI): 3.71, 13.73) and highest, but not middle, tercile of a wealth index. An association between MDE and hazardous drinking was not significant among the lowest, but was among the middle and highest, wealth tercile. Associations between MDE and hazardous or harmful-dependent drinking were both not significant among a lower education level, but were significant among the higher education level [30]. Mulia and Zemore [31] found that an association between depressive symptoms and heavy drinking was significant both among those with income below the federal poverty line (β = 0.24, *p* < 0.001), and above the federal poverty line [31]. Similarly, Martinez et al. [32] found significant associations between depressive symptoms and an increased risk of consuming five or more drinks per past year typical drinking occasion, compared to one to two drinks per past year typical drinking occasion, among individuals across three education tertiles. Once adjusted for demographics (e.g., age, gender), the association remained significant among the lowest (relative risk ratio (RRR) = 1.60, 95% CI: 1.13, 2.34, *p* ≤ 0.05) and middle, but not among the highest tertile. The same association for risk of consuming three to four drinks per past year typical drinking occasion was significant only among the middle (this was no longer significant once adjusted for demographics), and not among the lowest or highest, education tertile. Associations between depressive symptoms and an increased risk of having seven to 12 heavy episodic drinking episodes in the past year were not significant across education tertiles. The same association for risk of having 13 or more heavy episodic drinking episodes was only significant among the middle education tertile, although this was again no longer significant once adjusted for demographics [32].

##### Stress

Two quantitative studies investigated associations between stress and AU in lower SEP populations. Exposure to hurricane-related stressors [33] and exposure to general stressors [34] were investigated. Cerda et al. [33] found a positive association (reported by authors as marginally significant at *p* < 0.10) between exposure to post hurricane-related (Hurricanes Katrina and Rita in 2005) stressors and increased AU in individuals with a history of low income (estimate = 58.41, standard error (SE) = 31.45), but not among individuals with a history of middle income. There was also a significant positive association between pre-Katrina/Rita and Katrina/Rita-associated events and increased AU among individuals with less than high school education (estimate = 92.69, SE = 42.06, *p* < 0.05), but not those with high school education, low-income history, or middle-income history. However, an association between pre-Katrina/Rita and Katrina/Rita-associated events and the odds of binge drinking for a higher number of days in 2007 (controlled for the number of days binged in 2005) was not significant for individuals with a low or middle income history, or those with less than high school, or high school, education [33]. In addition, Grzywacz and Almeida [34] found a significant association between daily stressors and binge drinking for individuals with less than high school education (*b*=−0.63, SE = 0.09, *p* < 0.001), but not for individuals with high school, or a vocational/technical degree or some college, education. An association between stress pile-up and binge drinking was significant for individuals with less than high school education (*b* = 0.67, SE = 0.19, *p* < 0.001), but not for individuals with high school, or a vocational/technical degree or some college, education [34].

##### Adolescence

Two quantitative studies investigated associations between adolescence-related factors and AU in adulthood in lower SEP populations. Intense working hours during adolescence [35] and experiencing repeated alcohol intoxication during adolescence [36] were investigated. Rocheleau [35] found that lower social class background did not significantly moderate an association between invested work in adolescence and binge drinking likelihood among 22- to 25-year-olds or 27- to 31-year-olds. Higher social class background significantly moderated this association among 22 to 25 (*b*=−1.22, *p* < 0.05; SE = 0.32; marginal effect=−0.19, *p* < 0.05), but not among 27 to 31, year olds [35]. Whereas, Yaogo et al. [36] found significant associations between repeated adolescent alcohol intoxication and adulthood alcohol abuse among individuals from low-income families (OR = 11.86, CI: 3.35, 41.94, *p* = 0.0001), and individuals from intermediate or high-income families (OR = 2.49, CI: 1.09, 5.68, *p* = 0.02) [36].

##### Availability

Shortt et al. [37] found that total alcohol outlet density was significantly positively associated with AU probability for three out of four measures (exceeding guidelines, binge drinking, and problem drinking; all *p* < 0.05), but not harmful drinking (although there were significant increases for both on-sales (consumption on premises licenced to sell alcohol, e.g., a pub) and off-sales (alcohol for consumption away from the point-of-sale premise, e.g., a supermarket) outlet densities for harmful drinking; *p* < 0.05), among a low-income group. A high income group demonstrated non-significant increases for exceeding guidelines and binge drinking, and non-significant decreases for harmful drinking and problem drinking [37].

##### Cognitive factors

Sabia et al. [38] found a significant association between drinking more than 21 drinks a week and a reduced lower cognitive performance test score, than those who drank four to 14 drinks a week, in a low occupational group (unskilled workers) (95% CI: −3.9, −0.3, *p* < 0.05), and a low educational group (primary school education only) (95% CI: −7.1, −0.0, *p* < 0.05). This was also significant among those who drank one to three drinks a week in the low education group (95% CI: −8.8, −0.5, *p* < 0.05). Associations were not significant in the intermediate (professional qualification) and high educational (secondary school or higher), and intermediate (skilled worker) and high occupational (managers), groups for any AU levels [38].

#### Psychosocial pathways contributing to associations between AU and lower SEP

Eleven studies investigated psychosocial factor pathways associated with AU in lower SEP populations (see Table 4).

##### Stress, mental health, and drinking motives

Shuai et al. [39] found a significant indirect pathway (β = 0.012, SE = 0.006, CI = 0.003, 0.026) between socioeconomic deprivation and susceptibility to AU via aversive experience, internalizing symptoms and drinking to cope. This remained significant once demographic covariates were accounted for [39]. Similarly, Heim et al. [40] found that lower social grade was associated with greater reporting of coping drinking motives (*r* = 0.082, *p* < 0.01), whilst no other drinking motives were associated with social grade. Structural equation modelling indicated that social grade was significantly associated with coping drinking motives (standardised estimate = 0.06, *p* < 0.05), which were in turn significantly associated with AU (standardised estimate = 0.28, *p* < 0.001) [40]. Monk et al. [41] also found that lower social grade was significantly associated with increased coping drinking motives (estimate=−0.068, SE = 0.024, β=−0.039, *p* = 0.006), which were significantly associated with increased AU (estimate = 0.755, SE = 0.051, β = 0.232, *p* < 0.001). Social grade was not significantly associated with mental health, which was significantly associated with coping (estimate = 0.181, SE = 0.014, β = 0.213, *p* < 0.001) and conformity (estimate = 0.044, SE = 0.011, β = 0.065, *p* < 0.001) drinking motives (but not with social and enhancement drinking motives). Coping, social (estimate = 0.226, SE = 0.049, β = 0.088, *p* < 0.001) and enhancement (estimate = 0.957, SE = 0.049, β = 0.368, *p* < 0.001) drinking motives were significantly associated with increased AU, whereas conformity drinking motives (estimate=−0.385, SE = 0.063, β=−0.095, *p* < 0.001) were significantly associated with reduced AU [41].

##### Mental health and other social factors

Rodrigues-Stanley et al. [42] found non-significant indirect effects of SES and subjective social status on AU or AU change via psychological distress (comprising depressive cognition, depressive affect, and perceived stress) [42]. Whereas, Segrin et al. [43] found a significant indirect effect of neighbourhood disadvantage on problem drinking via psychological distress (comprising depression, perceived stress, loneliness) over a six month period (*b* = 6.588e − 7, 95% CI = 1.109e − 7, 1.329e − 6, β = 0.03, *p* < 0.05) [43]. Segrin et al. [44] also found a significant indirect effect of neighbourhood disadvantage on increased AU via negative emotionality (*b* = 0.02, 95% CI = 0.01, 0.04), SE = 0.01, *p* = 0.01), but a non-significant indirect effect of neighbourhood disadvantage on AU via social support [44].

##### Cognitive factors

McCarthy et al. [45] found a significant indirect effect of education on AU via positive alcohol expectancies (*Z*=–1.85, *p* < 0.05) [45].

##### Availability

Pollack et al. [46] found that alcohol availability did not significantly mediate an association between neighbourhood deprivation and the odds of heavy AU [46].

##### Other psychosocial factors

Zhu et al. [47] found that impulsivity did not significantly moderate an association between family economic hardship and risky AU [47]. Puddephatt et al. [48] found that compared to a higher SES class (professional occupation, degree-level educated, homeowners), there were significant indirect associations between a class of economically inactive, GCSE level or lower social renters and harmful/probable dependent drinking via social support (unstandardised coefficient (SE) = 0.23(0.09), 95% CI = 0.08, 0.39, *p* = 0.01) and neighbourhood environment (e.g., social cohesion) (unstandardised coefficient (SE) = 0.13(0.06), 95% CI = 0.03, 0.22, *p* = 0.03). An indirect effect of social support was also significant for another class (retired, no formal education, homeowners) (unstandardised coefficient (SE) = 0.06(0.03), 95% CI = 0.01, 0.11, *p* = 0.04). Compared to a higher SES class, there were no significant indirect associations between any other SES classes and hazardous drinking via social support or neighbourhood environment [48]. Elliott and Lowman [49] found that: (a) associations between higher education (β=−0.007, *p* < 0.05) and income (β=−0.005, *p* < 0.05) and reduced AU were mediated by external locus of control, (b) an association between higher income and reduced likelihood of meeting criteria for alcohol abuse or dependence over time was mediated by internal locus of control (β=−0.009, *p* < 0.01], c) an association between higher income and increased alcohol misuse was mediated by religiosity (β = 0.008, *p* < 0.01], and d) an association between higher income and increased likelihood of meeting criteria for alcohol abuse or dependence was mediated by religiosity (β = 0.006, *p* < 0.05). The authors report religiosity as a protective factor against, and external locus of control as a risk factor for, AU among lower income individuals [49].

#### Associations between AU and lower SEP, with a psychosocial factor adjusted for as a confounder

Three studies investigated associations between AU and lower SEP, tested with a psychosocial factor adjusted for as a confounder (see Table 5).

##### Mental health and other social factors

Two studies investigated whether a mental health factor may contribute to an association between AU and lower SEP. Boniface et al. [50] found that an association between socioeconomic group and risk of harmful or dependent AU among economically inactive renters with low education levels was significant when adjusted for confounders such as age and gender (RRR = 3.05, SE = 1.63, 95% CI: 1.07, 8.71, *p* = 0.037). However, this association was no longer significant when also adjusted for common mental disorder (RRR = 1.71, SE = 0.96, 95% CI = 0.57, 5.14, *p* = 0.335) [50]. In contrast, Grzywacz and Almeida [34] found that after adjusting for negative affect as a covariate in associations between binge drinking and (a) daily stressors (*b*=−0.42, SE = 0.05, *p* < 0.001) and (b) stress pile-up (*b* = 0.43, SE = 0.11, *p* < 0.001), significant associations remained for individuals with less than high school education [34]. Furthermore, Zhu et al. [47] found that an association between family economic hardship and risky AU was no longer significant once perceived discrimination was accounted for as a mediator (*b* = 0.09, *p* > 0.05) [47].

### Qualitative studies

Nine qualitative studies were included (see Table 6).

#### Drinking motives, stress, and mental health

Four qualitative studies outlined drinking motives, stress, and mental health factors related to AU in lower SEP populations [16, 51–53].

Lunnay et al. [51] found that working class women described AU as something enjoyable to look forward to, which enabled them to avoid thinking about negative occurrences in daily life and relieve loneliness and boredom. Experiencing a hangover was described as a way to occupy the day in the absence of limited other options, distract from daily life, and demonstrate control over their life. These factors, as well as a lack of social support, were highlighted as reducing inclinations to decrease AU [51]. Lunnay et al. [53] found that working class women described AU as a tool to cope with and remove stress, particularly in the context of alternative coping methods (e.g., social activities) being higher in cost. Alcohol was described as beneficial for their mental health, by enabling a sense of more control in their lives and bringing people closer together. They described drinking alcohol alone at home to cope with loneliness and stress [53]. Similarly, Lunnay et al. [16] found that working class women reported using alcohol to provide comfort and stress relief (in the absence of alternatives, such as secure employment). Alcohol was also reported to provide accessible support during difficult life events (e.g., losing a home), distract from loneliness, and act as a coping tool for stress [16]. In support, Ward et al. [52] found that working class women described using alcohol reactively as a means to manage stress in their lives, which resulted from personal and family stressors (e.g., health and financial issues). AU was seen to promote relaxation and wellness in the context of coping with stress. They described having less access to alternative means other than alcohol to enhance wellness (e.g., exercise classes). Reduced access to alternative means was described in terms of both financial access (e.g., classes were financially unaffordable), as well as reduced cultural and social access (e.g., feeling of not fitting in at classes) [52].

#### Other psychosocial factors

Five qualitative studies outlined additional psychosocial factors related to AU in lower SEP populations [54–58].

Arora et al. [54] found that differences in drinking behaviours between SES groups were explained via social acceptance of group drinking patterns, in which lower SES groups described heavy drinking and being drunk as socially acceptable. This was contrasted to the higher SES groups who described consuming a small amount of alcohol at one time, which was seen by this group as necessary for being presentable [54]. Järvinen [57] found that working class individuals described engaging in a pattern of occasional binge drinking (e.g., only weekend drinking), which they saw as less risky than daily drinking. This was contrasted with upper social class individuals who described drinking a little alcohol daily, with some combining this with occasional binge drinking. Working class individuals saw regular drinking (e.g., daily wine drinking) as riskier than middle- or upper-class individuals [57]. Molina de la Fuente et al. [56] found that residents of a lower socioeconomic district described drinking alcohol as enabling socialising with friends. Drinking socially with friends outside the home, at a bar or social event, was seen as common [56]. In a similar study, Molina de la Fuente et al. [55] found that residents of a lower socioeconomic district highlighted that drinking alcohol was linked to other addictive behaviours, such as gambling [55]. Hart [58] found that residents from disadvantaged communities described the influence of their family experience with alcohol on their drinking, in terms of either replicating or avoiding it. A family culture of occasional binge drinking was linked to frequent AU. Whereas a family and religious culture of not drinking alcohol was linked to feeling shame and conflicts with peer social norms for those who drank alcohol. Participants who had experienced traumatic alcohol-related events in their family described experiencing negative emotions and memories while drinking alcohol [58].

## Discussion

This scoping review synthesises the literature on psychosocial factors associated with AU in lower SEP populations to inform tailored interventions. Factors related to mental health, stress, drinking motives, alcohol availability, factors measured in adolescence, cognitive factors, and other psychosocial factors.

Four quantitative studies indicated that mental health may contribute to a relationship between lower SEP and AU [39, 43, 44, 50]. However, three quantitative studies found significant associations between mental health factors and AU among both lower and higher SEP populations [30–32], suggesting that mental health may be associated with AU across SEP. In support, individuals in the global general population with common mental health disorders (compared to those without) have twice the likelihood of having an alcohol use disorder [59]. However, rates of mental health disorders tend to be higher among lower SEP populations [60], suggesting that links between AU and mental health in lower SEP populations could be due to the elevated rate of mental health disorders in this demographic. In contrast, one qualitative study highlighted perceived mental health benefits from drinking in a lower SEP population [53]. However, AU in the general population has negative mental health impacts in the long-term [61]. Therefore, possible misconceptions about the long-term mental health benefits of drinking among lower SEP populations may represent a target for interventions. Interventions may also need to account for the perceived mental health benefits reported by lower SEP individuals as potential motivations for AU.

Stress may contribute to a potential pathway explaining the relationship between lower SEP and AU [39]. Two quantitative studies found that stress exposure is associated with AU among lower SEP populations [33, 34]. This supports the stressor-vulnerability model, which highlights that those most likely to drink in response to stress may have increased positive expectations of drinking and reduced resources to cope with stress [62]. A link between stress and AU among lower SEP populations may also be explained by the qualitative studies in this review. Two qualitative studies reported that AU among lower SEP populations was linked to having less access to alternatives (e.g., exercise classes) other than alcohol to enhance wellbeing [52], and that alternative coping methods (e.g., social activities) were more expensive than alcohol [53]. Stressors identified in this review varied from daily life stressors [34] to disaster-related stressors (e.g., hurricane) [33], which may reflect that AU-promoting stress may be derived from individual stressful events (e.g., divorce) and societal stressful events (e.g., terrorism) [63]. The COVID-19 pandemic is an example of a societal event linked to AU [64]. The impact of stress on AU may be amplified among lower SEP populations due to greater exposure to poverty-related stressors (e.g., finances, conflict, discrimination) [65]. Moreover, the quantitative studies indicated that coping drinking motives may contribute to an association between AU and lower SEP [39–41]. The qualitative studies highlighted that drinking motives include: distraction from daily life [51], coping with stress [16], coping with stress and improving mental health [53], and managing personal and family stressors [52]. This aligns with a motivational model which suggests that individuals drink when the perceived affective benefits exceed those of not drinking [66]. Consequently, interventions may need to account for lower SEP populations experiencing more stressors and different types of stressors, yet having reduced access to alternative coping resources other than alcohol for stress.

Findings for alcohol availability were mixed. One quantitative study found significant associations between density of alcohol outlets and AU for most drinking measures among lower SEP populations [37]. This supports an availability theory which proposes that increased alcohol availability is linked to increased AU and related harms [67]. Moreover, alcohol outlets are more likely to be located within disadvantaged districts [68]. However, another quantitative study found that alcohol availability did not significantly mediate an association between deprivation and AU [46]. This aligns with a review which indicated a lack of conclusive evidence on the relationship between alcohol outlet density and AU [69]. The lack of consensus may be explained by limitations of availability measurement, such as outlets often being categorised at a broad level (e.g., rather than, for example, supermarkets specifically), and a lack of consideration of online availability and individuals travelling to purchase alcohol [70]. Furthermore, a qualitative review of the impact of alcohol availability suggested that high availability not only increases exposure to alcohol, but may promote drinking in the absence of alternative community-based recreational activities [71]. This indicates that increased access to alcohol may exacerbate the use of alcohol as a coping strategy for stress in absence of access to alternative stress management methods in underserved communities, in line with the stress and coping drinking motive factors identified in this review.

One quantitative study found that increased AU was significantly associated with a lower cognitive test score among a lower SEP population [38]. However, AU also negatively impacts cognitive task performance among the general population [72], and it is difficult to determine causality between impaired cognition and AU in most studies [73]. Another quantitative study found that an association between AU and lower SEP was partly mediated by positive expectations of AU [45]. The mediating role of positive expected outcomes of drinking supports a social cognitive approach [74]. It may be that the particular stressors and reduced coping resources experienced by lower SEP populations lead this group to seek positive outcomes from drinking. Furthermore, findings for adolescence-related factors were mixed. One quantitative study found that repeated adolescent alcohol intoxication was associated with increased later alcohol abuse in adulthood among a lower SEP population [36]. Whereas another quantitative study found that an association between long working hours in adolescence and binge drinking was not significantly moderated by lower social class background [35]. An association between behavioural adolescence factors and later adult drinking reflects a life course perspective which highlights the importance of time periods in influencing AU [75]. Therefore, interventions for lower SEP adult drinking could incorporate a longitudinal perspective by targeting at-risk adolescents.

Furthermore, social and risk perceptions of drinking patterns were identified by two qualitative studies. A social acceptance of heavy drinking and being drunk among lower SEP individuals was identified by one qualitative study [54], and binge drinking being perceived as less risky than regular drinking was identified by another qualitative study [57]. However, evidence indicates that organ damage may be more likely to follow binge drinking than moderate drinking [76]. The influence of family drinking culture was identified by another qualitative study [58]. Evidence suggests a positive association between parental drinking and drinking in their children, although causality is unclear [77, 78].

Health interventions should be informed by factors associated with a health behaviour [7]. Public health interventions for AU in lower SEP groups could target the following areas: (1) poor mental health and perceived mental health benefits of drinking, (2) stress exposure, drinking response, and reduced alternative coping resources, (3) drinking motives and underlying motivations for drinking (e.g., expected positive outcomes), (4) alcohol availability, (5) adolescent behaviours, and (6) social and risk perceptions of different drinking patterns (e.g., heavy episodic drinking) and cultural family influence. This review indicates that individual motivations for AU to cope with stress and mental health may be compounded by the environmental context of living in underserved districts, and therefore having increased access to alcohol, but reduced access to alternative coping strategies. This supports explanations of the alcohol harm paradox which suggest that lower SEP populations have reduced access to protective resources for coping with stress [4]. Policymakers implementing interventions for AU could consider not only why lower SEP populations drink alcohol on an individual level (e.g., stress), but how this is impacted by the unequal balance of low availability of alternative coping and wellbeing resources (e.g., exercise classes [52]) and high availability of alcohol [37] at a community level. For example, recommended interventions to reduce availability of and access to alcohol [79] may also need to increase availability of and access to alternative stress management resources (e.g., studies in this review highlight reduced access to social activities [53] and exercise classes [52]) in underserved communities to increase intervention acceptability among lower SEP populations. Future research could investigate what kinds of alternative community resources to support wellbeing would be acceptable to lower SEP populations.

### Strengths and limitations

This review provides insight into the range of psychosocial factors associated with AU in lower SEP populations. The use of a scoping review design and incorporation of a psychosocial approach to associated factors enabled mapping of a broad research area [21].

However, this review should be considered with the following limitations in mind. Lower SEP measurement and ranges within studies varied across studies, which limits comparisons. However, a broad approach was necessary due to the variety of measures in the literature, and to meet our objective to scope the existing literature. Guidelines on SEP measurement highlight that there may not be one optimal indicator due to differing underlying theoretical bases of each indicator [80]. Other reviews on socioeconomic health inequalities have also incorporated the range of available measures [81–83]. Use of a single SEP measure may have excluded relevant studies, as income and education indicators tend to be preferred in the USA, whereas occupational indicators tend to be preferred in Europe [84]. Many studies were correlational. We therefore cannot infer causality between most factors and AU, and variables not measured in these studies may have explanatory value. Whilst the lack of quality appraisal or risk of bias assessment aligns with scoping review methodology, it does limit the interpretability of reported associations. Whilst our synthesis of reporting significant associations aligns with other scoping reviews which have followed this approach [23–25], the lack of quality appraisal notably hinders inferences which can be drawn. We therefore suggest that implications of findings of this scoping review should be validated by further systematic review and quality assessment to enable strong recommendations for interventions. The incorporation of a broad set of psychosocial factors [21] may also hinder interpretation of findings to some extent. Moreover, AU measurement varied, again limiting comparability. Most studies used self-report measures, and AU tends to be under-reported [85]. The studies identified within this review were also dependent on the search strategy used, informed by the psychosocial approach. Therefore, we may not have fully captured insights from studies beyond the boundaries of our defined search terms, such as studies which focused on more structural and policy level factors linked with AU. The quantitative studies identified were also dependent on statistical associations being investigated, which may arguably be harder to examine for structural factors affecting AU. We also did not search the grey literature.

## Conclusions

We identified an array of psychosocial factors associated with AU in lower SEP populations which could inform the direction of future research for the development of tailored interventions. The evidence base is predominantly comprised of quantitative studies investigating factors such as mental health and stress. Future research in the area would benefit from greater use of qualitative studies to complement these insights and generate improved understanding of experiences of AU among lower SEP populations. This may advance knowledge of the alcohol harm paradox and its potential mechanisms. Taken altogether, the findings of this scoping review suggest that individual-level drinking motivations (e.g., coping with stress and mental health, positive expected outcomes, risk perception of drinking patterns) may be amplified by structural neighbourhood designs that increase availability of alcohol as a coping tool, yet reduce access to alternative coping strategies. Therefore, recommended population level alcohol availability and access reduction intervention approaches [79] could concurrently integrate improved access to alternative stress management resources in underserved communities to increase the acceptability of such interventions among lower SEP populations.

## Supplementary Information

Below is the link to the electronic supplementary material.

Supplementary Material 1

Supplementary Material 2

## Data Availability

All data are publicly available.

## Abbreviations

AU: Alcohol use
SEP: Socioeconomic position
MUP: Minimum unit pricing
SES: Socioeconomic status
MDE: Major depressive episode
OR: Odds ratio
CI: Confidence interval
RRR: Relative risk ratio
SE: Standard error
