# Supplementary material for: Psychosocial factors associated with alcohol use in lower socioeconomic position populations: a scoping review
Source: BMC Public Health. 2025 Oct 31;25:3676. doi: 10.1186/s12889-025-24508-z (PMC12577420; doi:10.1186/s12889-025-24508-z)
Supplement: Supplementary file 1 — Supplementary Material 1 [file 12889_2025_24508_MOESM1_ESM.docx]

**Supplementary Materials for Psychosocial factors associated with alcohol use in lower socioeconomic position populations: A scoping review**

Literature searches were carried out on 19^th^ December 2022. The following search string was used: (alcohol*) AND (influenc* or predict* or determin* or associat*) AND (socio-economic* or socioeconomic* or “social class” or disadvantag*) AND (psycho* or social*) NOT (tobacco* or smok*). The following five databases were searched: Web of Science (Core Collection), Scopus, Embase (Embase and Medline), PubMed, and APAPsycNet (PsycInfo). The search was limited to articles published from 1^st^ January 2000 to the date of search in order to identify factors relevant to contemporary society.

**Full search strategy**

**Web of Science**

**Search string:**

TS=(alcohol*) AND TS=(influenc* or predict* or determin* or associat*) AND TS=(socio-economic* or socioeconomic* or “social class” or disadvantag*) AND TS=(psycho* or social*) NOT TS=(tobacco* or smok*)

**Database restrictions:**

Web of Science (Core Collection, all editions).

**Time restrictions:**

1^st^ January 2000 to date of search.

**Filter restrictions:**

Results will be filtered by “review article” for exclusion.

**Scopus**

**Search string:**

TITLE-ABS-KEY =(alcohol*) AND TITLE-ABS-KEY=(influenc* or predict* or determin* or associat*) AND TITLE-ABS-KEY=(socio-economic* or socioeconomic* or “social class” or disadvantag*) AND TITLE-ABS-KEY=(psycho* or social*) NOT TS=(tobacco* or smok*)

**Time restrictions:**

2000 to present.

**Filter restrictions:**

Results will be filtered by “review” for exclusion.

**Embase**

**Search string:**

ti,ab,kw=(alcohol*) AND ti,ab,kw=(influenc* or predict* or determin* or associat*) AND ti,ab,kw=(socio-economic* or socioeconomic* or “social class” or disadvantag*) AND ti,ab,kw=(psycho* or social*) NOT ti,ab,kw=(tobacco* or smok*)

**Database restrictions:**

Embase (Embase and Medline)

**Time restrictions:**

2000 to 2022.

**Filter restrictions:**

Results will be filtered by all publications types for inclusion, except for “review” (in order to exclude review articles).

**PubMed**

**Search string:**

Textword=(alcohol*) AND Textword=(influenc* or predict* or determin* or associat*) AND Textword=(socio-economic* or socioeconomic* or “social class” or disadvantag*) AND Textword=(psycho* or social*) NOT Textword=(tobacco* or smok*)

**Time restrictions:**

2000 to 2022.

**Filter restrictions:**

Results will be filtered by all publications types for inclusion, except for “review” and “systematic review” (in order to exclude review articles).

**APA PsycNet**

**Search string:**

Title=(alcohol*) AND TS=(influenc* or predict* or determin* or associat*) AND Title=(socio-economic* or socioeconomic* or “social class” or disadvantag*) AND Title=(psycho* or social*) NOT Title=(tobacco* or smok*)

Abstract=(alcohol*) AND Abstract=(influenc* or predict* or determin* or associat*) AND Abstract=(socio-economic* or socioeconomic* or “social class” or disadvantag*) AND Abstract=(psycho* or social*) NOT Abstract =(tobacco* or smok*)

Index Terms=(alcohol*) AND Index Terms=(influenc* or predict* or determin* or associat*) AND Index Terms=(socio-economic* or socioeconomic* or “social class” or disadvantag*) AND Index Terms=(psycho* or social*) NOT Index Terms=(tobacco* or smok*)

Keywords=(alcohol*) AND Keywords=(influenc* or predict* or determin* or associat*) AND Keywords=(socio-economic* or socioeconomic* or “social class” or disadvantag*) AND Keywords=(psycho* or social*) NOT Keywords=(tobacco* or smok*)

**Database restrictions:**

APA PsycNet (PsycInfo).

**Time restrictions:**

2000 to 2022.

**Filter restrictions:**

Results will be filtered by “methodology is not literature review or systematic review”.
